# Congenital quadricuspid aortic valve associated with aortic insufficiency and mitral regurgitation

**DOI:** 10.1186/1749-8090-8-87

**Published:** 2013-04-15

**Authors:** Jiaquan Zhu, Junwen Zhang, Shubin Wu, Yunjiao Zhang, Fangbao Ding, Ju Mei

**Affiliations:** 1 Cardiovascular Surgery Division, Cardiac Center, Xinhua Hospital, School of Medicine, Shanghai Jiaotong University, 1665 Kongjiang Road, Shanghai 200092, China

**Keywords:** Quadricuspid aortic valve, Aortic regurgitation, Surgery

## Abstract

Congenital quadricuspid aortic valve is a rare cardiac anomaly. More than half of the patients with this abnormality will develop aortic insufficiency in adulthood. It is vital that patients with quadricuspid aortic valve who present with progressive aortic regurgitation undergo valve replacement or repair at appropriate time. Here, we present two cases of quadricuspid aortic valve. We first describe a 58-year-old man who had mitral regurgitation and ascending aorta dilatation with quadricuspid aortic valve. He underwent aortic valve replacement and mitral valve plasty and recovered well. The second patient is a 20-year-old asymptomatic boy who has been closely followed up and has not received any surgical treatment.

## Background

Among congenital aortic valve anomalies, quadricuspid aortic valve (QAV) is rare relative to the more common bicuspid aortic valve lesion. More than half of these patients need valve surgery in adulthood mainly due to progressive aortic regurgitation. Here, we present two cases of QAV with one surgical patient, and the other non-surgical.

## Case presentation

### Case 1

**Figure 1:**
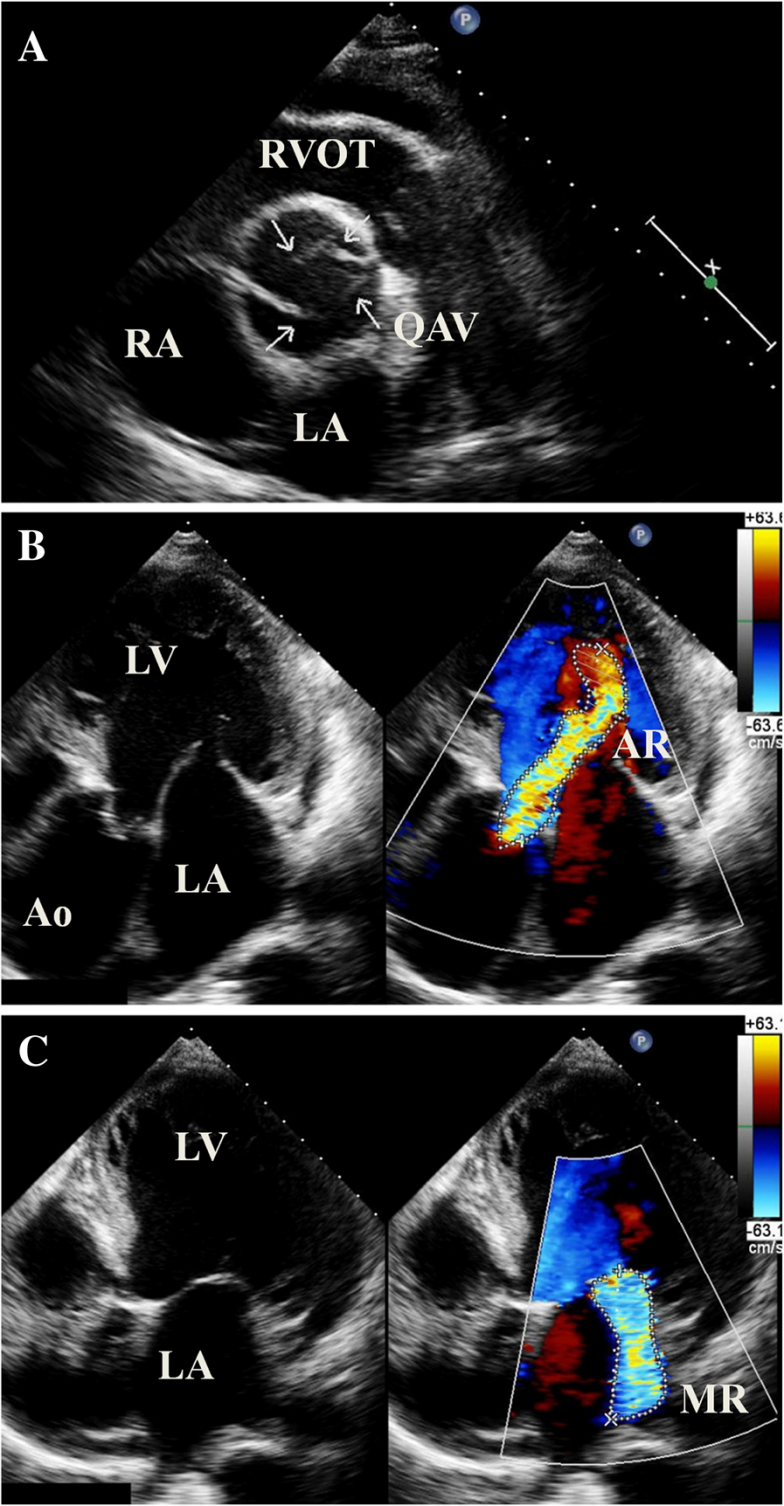
**Echocardiography of a 58-year old man with quadricuspid aortic valve (QAV). **Figure 1A, short axis view shows the four cusps of aortic valve; Figure 1B, severe aortic regurgitation and dilated aortic root; Figure 1C, mitral regurgitation. RVOT, right ventricular outflow tract; RA, right atrium; LA, left atrium; LV, left ventricle; Ao, Aorta; AR, aortic regurgitation; MR, mitral regurgitation.

**Figure 2:**
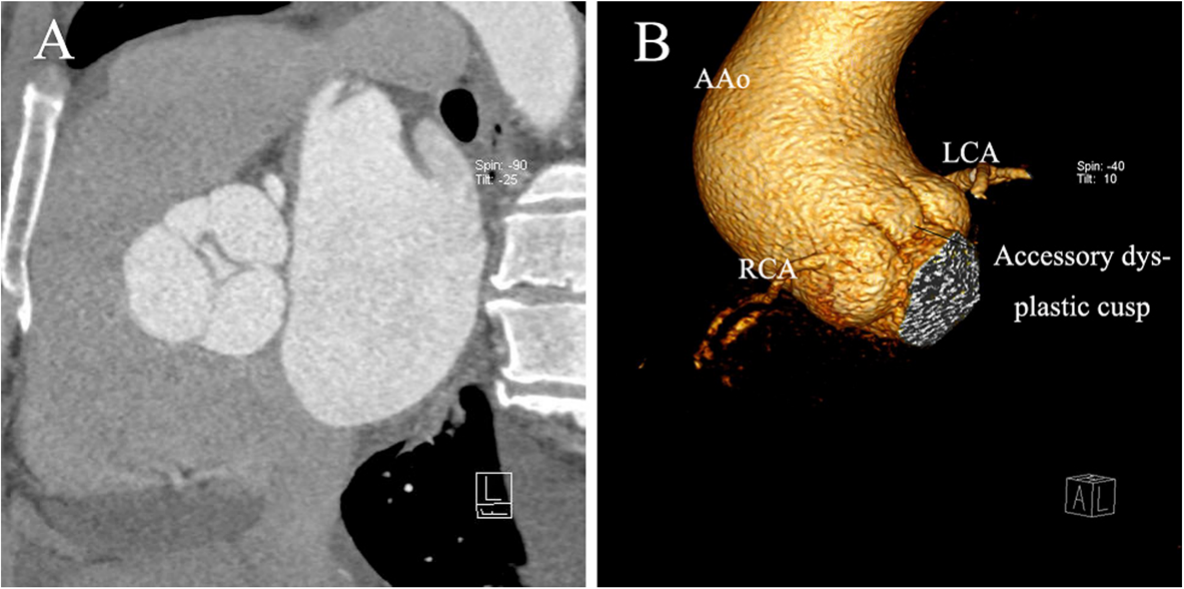
**Computerized tomography angiogram of a quadricuspid aortic valve (QAV) patient. **Figure 2A, three normal cusps and one accessory dysplastic cusp with central aortic regurgitation (AR); Figure 2B, the dysplastic cusp located between left and right coronary sinuses, and ascending aorta (AAo) was dilated. LCA, left coronary artery; RCA, right coronary artery.

A 58-year-old Chinese man was admitted to our department with chief complaint of exertional dyspnea for one month. His past medical history was unremarkable. Upon physical examination, his apical pulse was enhanced and displaced to the lateral and caudal side, and auscultation revealed a grade four diastolic murmur at the left sternal border. Chest x-ray revealed cardiomegaly to left and inferior and an electrocardiogram indicated left ventricular hypertrophy. Transthoracic echocardiography in the short axis view revealed four aortic cusps (Figure 1A) with poor coaptation in diastole and caused severe central aortic regurgitation (Figure 1B). The left ventricle was significantly dilated with contractile dysfunction. The left ventricle end-diastolic diameter (LVEDD) was 74 mm, and the end-systolic diameter (LVESD) was 61 mm. Ejection fraction was 35%. Besides this, moderate to severe mitral valve regurgitation was found with a slightly enlarged left atrium (Figure 1C). The diameter of the aortic annulus was within normal range (29 mm); however, the aortic sinus and ascending aorta were dilated (Figure 1B, 44 mm). Mild tricuspid regurgitation and minimal pericardial effusion was also found. Contrast enhanced multi-detector dual source computerized tomography also show four separated aortic cusps (Figure 2A) with a dilated ascending aorta (Figure 2B).

This patient received medical therapy for a few weeks, and then underwent surgery. In the operating room, the echocardiography findings were confirmed. The four cusps consisted of three normally developed valves and an accessory dysplastic cusp, which was located between the right coronary and left coronary sinus. The coronary ostiums were normal, and the aortic valves were found to be thickened. We replaced the aortic valve with a 25 mm bioprosthesis (Edwards lifesciences). We also performed mitral annuloplasty with a 28 mm ring (Sorin). Intra-operative transesophageal echocardiography (TEE) indicated trace mitral regurgitation and good aortic valve function. Post-operative recovery was uneventful except for the use of intra-aortic balloon pump assistance for a few days. Before discharge, echocardiography demonstrated reduced left ventricle size (LVEDD 56 mm) and reasonable systolic function.

### Case 2

**Figure 3:**
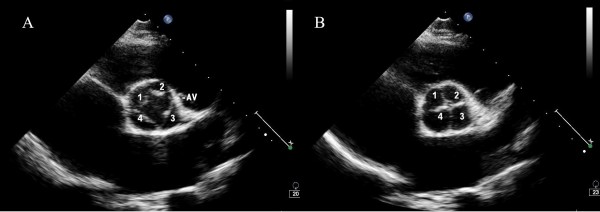
**Echocardiography of a 20-year-old asymptomatic boy with quadricuspid aortic valve (QAV).** Short axis view during systole (**A**) and diastole (**B**) revealed four equal cusps of aortic valve.

A 20-year-old asymptomatic Chinese boy was found to have grade 3/6 diastolic murmur at the left sternal border. Echocardiography revealed quadricuspid aortic valve with four equal cusps (Figure 3). There was moderate aortic insufficiency and mild to moderate mitral regurgitation. Left ventricle size was within normal range (LVEDD 53 mm, LVESD 32 mm), and ejection fraction was 70%. We opted for close follow up.

## Discussion

A normal aortic valve is composed of three symmetric cusps. Abnormal cusps may be formed as a result of a developmental anomaly during embryological arterial trunk septation. The most common type of aortic valve deformation is the bicuspid valve, followed by unicuspid valve. QAV is very rare and it is also less common than quadricuspid pulmonary valve [1]. QAV was reported to have an incidence of around 0.013~0.043% according to autopsy results [2]. QAV also accounted for 0.55~1.46% of aortic valve surgical patients [3,4]. In our center, these two patients were the only two in the last six years.

Before echocardiography was widely used three decades ago, most QAVs were diagnosed during surgery or autopsy [1] and seldom by aortography [5]. In recent years, the majority of QAV patients were diagnosed by non-invasive methods, with echocardiography being the most popular. On short axis view, QAV has an “X” shape in diastole, instead of a normal “Y” pattern (as shown in Figure 3B). Since transesophageal echocardiography is now widely used, we can get better images than by using transthoracic echocardiography [6,7]. Computerized tomography and MRI also serve as alternative diagnostic methods [8-10]. Unfortunately, we did not perform TEE preoperatively for the surgical patient in this study, because we were already sure about the QAV diagnosis, which was confirmed later both by computer tomography and inspection during the operation.

According to the anatomy of the four cusps, Hurwitz and Roberts categorized QAV into seven subtypes (A to G) [11]. The two most frequent types are type A (four equal cusps) and type B (three normal cusps with one smaller cusp). It is thought that type B has a greater probability of developing aortic regurgitation because of the asymmetric shear stress of the cusps. Our first case belonged to type B with severe aortic insufficiency, while the second case was type A. Recently, Jagannath et al. summarized a detailed literature review and simplified the classification of QAV (type I to type IV) [12]. Type I and type II are the same as the previously described types A and B.

Unlike the relatively stable quadricuspid pulmonary valve anatomy, more than half of QAV patients developed aortic regurgitation progressively with aortic stenosis seldom seen. Most of them need surgery in their fifties to sixties [12]. Except for the possible subsequent lesion of aortic regurgitation, some QAVs are often associated with other abnormalities, such as displacement of the coronary sinus and ostium, ventricular septal defect, patent duct arteriosus, subaortic stenosis, cardiomyopathy, valsalva aneurysm, and mitral valve regurgitation [13-15]. Although Jagannath et al. indicated that aortic root dilatation was rare in QAV patients in their latest review, aortic root aneurysm might occasionally occur, as in our case and also reported by others [16-18]. This may be a result of long-term aortic regurgitation. Left ventricular dysfunction was also occasionally seen in some cases, and the surgical outcomes for those cases were satisfactory [19,20], as seen in our first patient who had low ejection fraction pre-operatively but recovered well with temporary assistance of an intra-aortic balloon pump.

The surgical indication of QAV patients depends on the extent of aortic regurgitation and its associated lesions. For aortic regurgitation, the indication is almost the same as regurgitation caused by other reasons, such as degenerative disease. However, surgeons should pay attention to the origin of the coronary artery, and avoid injury when operating. As aortic valve repair is still a big challenge for cardiac surgeons, valve replacement was the most widely used operation in such patients. Some surgeons have tried tri-cuspidized or bi-cuspidized repair techniques and obtained good short-term results [9,21-23]. These patients can avoid complications associated with valve replacement, but long-term follow up is required.

## Conclusions

In summary, we presented two cases of QAV in this paper. The first patient had severe aortic regurgitation resulting from a quadricuspid aortic, along with mitral regurgitation and ascending aorta dilatation. The second was a non-surgical asymptomatic young patient. With the advancement of echocardiography and multi-detector computer tomography, QAV has been diagnosed more accurately and promptly in recent years. Valve replacement or repair is needed at appropriate time in patients with progressive aortic regurgitation.

## Consent

Written informed consent was obtained from the patients for publication of this Case Report and any accompanying images. A copy of the written consent is available for review by the Editor-in-Chief of this journal.

## Abbreviations

QAV: Quadricuspid aortic valve; LVEDD: Left ventricle end-diastolic diameter; LVESD: Left ventricle end-systolic diameter; TEE: Transesophageal echocardiography.

## Competing interests

The authors declare that they have no competing interests.

## Authors’ contribution

JZ and JZ collected the data and prepared the manuscript; SW performed and provided the ECHO data; YZ, FD, and JM operated on the patient and revised the manuscript. All authors read and approved the final manuscript.
